# Understanding the Molecular Epidemiology and Global Relationships of *Brachyspira hyodysenteriae* from Swine Herds in the United States: A Multi-Locus Sequence Typing Approach

**DOI:** 10.1371/journal.pone.0107176

**Published:** 2014-09-05

**Authors:** Nandita S. Mirajkar, Connie J. Gebhart

**Affiliations:** 1 Department of Veterinary and Biomedical Sciences, College of Veterinary Medicine, University of Minnesota, Saint Paul, Minnesota, United States of America; 2 Veterinary Diagnostic Laboratory, College of Veterinary Medicine, University of Minnesota, Saint Paul, Minnesota, United States of America; Naval Research Laboratory, United States of America

## Abstract

Outbreaks of mucohemorrhagic diarrhea in pigs caused by *Brachyspira hyodysenteriae* in the late 2000s indicated the re-emergence of Swine Dysentery (SD) in the U.S. Although the clinical disease was absent in the U.S. since the early 1990s, it continued to cause significant economic losses to other swine rearing countries worldwide. This study aims to fill the gap in knowledge pertaining to the re-emergence and epidemiology of *B. hyodysenteriae* in the U.S. and its global relationships using a multi-locus sequence typing (MLST) approach. Fifty-nine post re-emergent isolates originating from a variety of sources in the U.S. were characterized by MLST, analyzed for epidemiological relationships (within and between multiple sites of swine systems), and were compared with pre re-emergent isolates from the U.S. Information for an additional 272 global isolates from the MLST database was utilized for international comparisons. Thirteen nucleotide sequence types (STs) including a predominant genotype (ST93) were identified in the post re-emergent U.S. isolates; some of which showed genetic similarity to the pre re-emergent STs thereby suggesting its likely role in the re-emergence of SD. In the U.S., in general, no more than one ST was found on a site; multiple sites of a common system shared a ST; and STs found in the U.S. were distinct from those identified globally. Of the 110 STs characterized from ten countries, only two were found in more than one country. The U.S. and global populations, identified as clonal and heterogeneous based on STs, showed close relatedness based on amino acid types (AATs). One predicted founder type (AAT9) and multiple predicted subgroup founder types identified for both the U.S. and the global population indicate the potential microevolution of this pathogen. This study elucidates the strain diversity and microevolution of *B. hyodysenteriae*, and highlights the utility of MLST for epidemiological and surveillance studies.

## Introduction

Swine dysentery (SD) is a mucohemorrhagic diarrheal disease of pigs caused by an anaerobic spirochete, *Brachyspira hyodysenteriae* [1]. The pathogen primarily affects the colon and cecum of grower-finisher pigs, although it can affect pigs of all ages [2]. Significant economic losses occur worldwide due to decreased growth rates, poor feed conversion, high morbidity and mortality, and the costs involved in treatment, prevention and control of SD in swine herds [3].

*B. hyodysenteriae* largely disappeared from the U.S. in the 1990s, although it continued to cause production losses to other swine-rearing countries globally. This disappearance of SD in the U.S. is thought to be due to changes in husbandry practices that reduced the transmission of the pathogen and consequently the detection of clinical disease. The disease re-emerged in the U.S. since the late 2000s, and it is hypothesized that the re-emergence is due to factors such as increased virulence of the pathogen, decreased susceptibility of the pathogen to antimicrobials or changes in feed/diet. Despite the economic importance of the disease in the U.S., no information currently exists on the epidemiology of this important pathogen within the U.S., or on the potential relatedness of these post re-emergent isolates (after the late 2000s) with those present in the U.S. pre re-emergence (before the early 1990s). Phenotypic features such as virulence [4] and antimicrobial susceptibility [5] vary in different strains and may play a role in this re-emergence, highlighting the importance of strain characterization.

Strain typing of *B. hyodysenteriae* has been conducted by various methods including restriction endonuclease analysis (REA) [6], [7], random amplification of polymorphic DNA (RAPD) [8], restriction fragment polymorphism (RFLP) [9] and pulsed field gel electrophoresis (PFGE) [10]–[12]; however these gel-based molecular typing methods have limited discriminatory power. Multilocus enzyme electrophoresis (MLEE) [13], [14] was found useful in studying the population structure of *B. hyodysenteriae*; however the technique is laborious and the results cannot be easily compared between laboratories. Multiple-locus variable-number tandem-repeat analysis (MLVA) is a high-resolution method that was found to be useful for local epidemiological studies [15]; however, the technique requires capillary electrophoresis for inter-laboratory comparability of the results.

Maiden and colleagues [16] used the unambiguous nature and the electronic portability of nucleotide sequence data to develop a unique technique known as multilocus sequence typing (MLST) for characterizing strains of microorganisms, which overcame many of the limitations associated with gel-based techniques. The technique utilizes the allelic variation in nucleotide sequences of multiple conserved housekeeping gene loci to differentiate strains of pathogens, and has proven very useful in global epidemiological studies of numerous infectious agents [17]. Osorio and colleagues [18] analyzed *B. hyodysenteriae* isolates using a *B. hyodysenteriae* species-specific MLST scheme developed by La and colleagues [19] which was based on modifications made to a preliminary *Brachyspira* genus-specific MLST scheme [20]. This MLST scheme is universally available (http://pubmlst.org/bhyodysenteriae/) for strain comparisons and epidemiological studies of *B. hyodysenteriae*.

The current study used the *B. hyodysenteriae* species-specific MLST scheme [19] to meet the following objectives: (1) To characterize and to investigate the diversity, distribution, microevolution and population structure of post re-emergent *B. hyodysenteriae* strains currently circulating in U.S. swine herds. (2) To compare the strain relatedness of the recently isolated strains (post re-emergence) with those isolated before the early 1990s (pre re-emergence). (3) To expand knowledge of the global epidemiology, population structure and microevolution of *B. hyodysenteriae*. (4) To elucidate the relationships of U.S. origin isolates with global *B. hyodysenteriae* isolates.

## Materials and Methods

### *B. hyodysenteriae* isolates

A diverse set of 69 *B. hyodysenteriae* isolates originating from a variety of geographical locations across North America from the 1970s to 2010s were evaluated in this study. Of these, 59 *B. hyodysenteriae* isolates were obtained from the frozen culture collection at the University of Minnesota Veterinary Diagnostic Laboratory (UMN-VDL). These isolates were confirmed to be *B. hyodysenteriae* if they showed strong hemolysis on blood agar cultures and also tested positive by a *B. hyodysenteriae*-specific PCR or by *nox* gene sequence analysis [21]. These isolates were selected to create a dataset that is representative of strains currently circulating in the U.S. and included isolates from all available sites (43 sites, 17 swine production systems and nine U.S. states). A swine ‘site’ refers to the swine farm from which the isolates originate and a swine ‘system’ refers to the production company that owns multiple such swine sites. Generally each site was represented by one isolate; however a selected subset of eight sites (from diverse systems) were represented by three isolates each (at a single time-point) to enable an intra-site analysis. Of the 69 isolates, data for the remaining 10 previously analyzed isolates [19] were obtained from the PubMLST database (http://pubmlst.org/bhyodysenteriae/). These isolates originated from North America (seven U.S. and three Canada) between the 1970s to the 1990s and included the ATCC type strain B78^T^ (ATCC 27164) as well as reference strains B204^R^ (ATCC 31212) and B234^R^ (ATCC 31287). Although most isolates in this collection originated from commercial pigs, one originated from a mouse on a swine site, and another from a rhea (Table S1).

A spatially and temporally diverse set of 341 *B. hyodysenteriae* isolates originating from Australia (24.05%), Europe (55.72%) and North America (20.23%) over five decades (1970s to 2010s) was generated. In addition to the 69 North American isolates, this included data for 272 *B. hyodysenteriae* isolates originating from countries outside of North America, obtained from the PubMLST database (http://pubmlst.org/bhyodysenteriae/), much of which was previously analyzed by MLST [18], [19]. The complete dataset of 341 isolates originated from 10 countries including Australia (n = 82), Belgium (n = 1), Canada (n = 3), Germany (n = 3), Italy (n = 108), Portugal (n = 1), Spain (n = 50), Sweden (n = 10), U.K. (n = 17) and the U.S. (n = 66). Most isolates originated from pigs; however some also originated from mice (n = 2), mallards (n = 2) and rhea (n = 1).

### Multi-locus sequence typing

The *B. hyodysenteriae* isolates were grown on tryptic soy agar plates with 5% defibrinated sheep blood and passaged at least twice from the freezer before being used for DNA extraction. The commercial sheep blood (I-Tek Medical Technologies, MN, USA) used above was not obtained from or used in any in vivo/animal experimental study, and therefore did not require approval from IACUC. The PrepMan Ultra sample preparation reagent (Applied Biosystems) was used to extract chromosomal DNA using the culture plate protocol as per manufacturer's instructions. Seven conserved housekeeping genes including alcohol dehydrogenase (*adh*), alkaline phosphatase (*alp*), esterase (*est*), glutamate dehydrogenase (*gdh*), glucose kinase (*glpK*), phosphoglucomutase (*pgm*) and acetyl-coA acetyltransferase (*thi*) were used as MLST loci as described previously [19]. The HotStarTaq Master Mix Kit (Qiagen, Valencia, CA, USA) was utilized to perform PCRs of housekeeping genes of the selected isolates, a positive control (*B. hyodysenteriae* strain WA1^R^) and a negative control (ultrapure water) as per the previously established protocol [19].

The PCR products were purified with QIAquick PCR Purification Kit (Qiagen, Valencia, CA, USA) as per the manufacturer's instructions. The purified PCR products were sequenced bi-directionally using the ABI 3730×l (96 Capillary) DNA analyzer (Applied Biosystems, San Francisco, CA, USA) at the University of Minnesota Genomics Center. The chromatograms were verified for good quality, and the sequences for each locus were aligned with that of the positive control *B. hyodysenteriae* strain WA1^R^ sequence [19] using ClustalW [22] in MEGA 5 [24] to ensure that the sense strand was used for analysis. The aligned loci sequences were trimmed to a previously described length and region [19] and the resultant DNA sequences were translated to predicted amino acid sequences using MEGA 5 [23].

### Analyses

The analysis was first conducted on the data obtained from isolates originating from North America, and this was followed by an analysis on data obtained from all isolates in the global dataset. The trimmed sequences of each locus were queried on the PubMLST Bacterial Isolate Genome Sequence Database (BIGSdb) (http://pubmlst.org/perl/bigsdb/bigsdb.pl?db=pubmlst_Brachyspira_seqdef) to identify any previously assigned alleles [24], and any unique nucleotide sequences identified were assigned unique allele numbers. The allelic profile of each isolate was determined by the combination of alleles at the seven loci. Isolates with allelic profiles matching those of a previously assigned sequence type (ST) were allotted its ST, and each isolate with a unique profile was assigned a new sequence type (ST). Isolates were considered to be of the same strain or ST if they were genetically identical at each of the seven loci. The same analysis protocol was applied to amino acid sequences and an amino acid type (AAT) was assigned to each isolate. The ST and AAT data for previously analyzed global isolates was obtained from the PubMLST database (http://pubmlst.org/bhyodysenteriae/) and from previous studies [18], [19]. Isolates were analyzed on four levels: within a site, between multiple sites within a system, within a state and globally (between countries).

The North American and global *B. hyodysenteriae* isolates were analyzed for relatedness by the Based Upon Related Sequence Types (BURST) and the Minimum Spanning Tree (MST) algorithms; and for recombination and diversity by the Index of association (I_A_) and by Simpson's Index of Diversity (DI) respectively. The BURST algorithm of eBURST v3 program was used to group isolates into clonal complexes (CCs) of single locus variants (SLVs) and to identify predicted founders and subgroup founders [25]. In addition, a population snapshot of STs was obtained by setting the minimum number of identical loci for a group definition to 0/7. The MST algorithm was used to group isolates into clonal complexes (CCs) of double locus variants (DLVs) and to create a network depicting the spatial and temporal distribution and relatedness of isolates using Bionumerics v7.1 software. A strict definition of allelic profiles differing at only one locus (SLVs) was used to define members of a CC by the BURST algorithm; however a more relaxed definition of allelic profiles differing at two loci (DLVs) was used to depict relatedness and potential microevolutionary descent by the MST algorithm. The same procedure of analysis was applied to both STs and AATs. To estimate the effect of recombination, the START2 program was used to calculate the Index of Association (I_A_) for the North American, Australian, Spanish and the combined global populations, using isolates and STs separately [26]. The mean ratio of non-synonymous to synonymous substitutions at the seven loci was calculated using START2, to estimate the selective pressure acting on the isolates from North America, Australia and Spain individually as well as the combined global populations [27]. Simpson's Index of Diversity was calculated for the North American population and the combined global population as previously described [28]. Dendograms to depict relatedness of isolates were conducted on the concatenated nucleotide and amino acid sequences of North American isolates, global STs and global AATs following the same procedure as described previously [18], [19].

## Results

### North American isolates

The 69 *B. hyodysenteriae* North American isolates represented 22 STs and 17 AATs based on the analyses of seven loci (Table S1). Of the 59 contemporary/post re-emergence isolates from the 2010s, 57 of them were represented by 12 unique and newly described STs. In comparison, the 10 historic/pre re-emergence isolates from the 1970s– early1990s were represented by nine unique and previously assigned STs [19]. Two contemporary U.S. isolates were characterized as ST56, the same ST as that of the historic ATCC type strain B78^T^ (ATCC 27164) which originated from the U.S. in the 1970s. With two exceptions (ST107 and ST110), all newly described STs contained at least one newly assigned allele. Table S1 contains details of allele numbers, STs and AATs for all North American isolates evaluated. Of the 17 AATs, seven newly described and five previously described AATs were unique to North America. A total of seven AATs were newly described, while three previously described AATs for historical North American isolates were also represented in the contemporary isolates. The complete collection of 69 isolates showed mean nucleotide and amino acid allelic frequencies over the seven loci of 7.43 and 3.29, respectively. The most frequently isolated strains in this dataset were ST93 and AAT9 based on nucleotides and amino acids, respectively. A total of 4,086 nucleotides were analyzed for each isolate, for which 73 (1.79%) variable positions were identified. Table 1 contains details of the mean G+C content, mean dN: dS ratio, DI value and I_A_ value.

**Table 1:** Summary numerical values of *B. hyodysenteriae* isolates and STs on a country and global level.

Level of discrimination	I_A_ (p-value)	mean dN/dS	mean% GC	DI
Global isolates	1.11 (p<0.0001)	0.091	34.89	0.920
Global STs	0.40 (p<0.0001)	n/a	34.87	n/a
North American isolates	2.55 (p<0.0001)	0.062	34.86	0.897
North American STs	0.30 (p = 0.0050)	n/a	34.88	n/a
Australian isolates	1.34 (p<0.0001)	0.108	34.84	0.977
Australian STs	1.07 (p<0.0001)	n/a	34.84	n/a
Spanish and Portuguese isolates	2.95 (p<0.0001)	0.087	34.88	0.758
Spanish and Portuguese STs	0.55 (p = 0.0280)	n/a	34.88	n/a
Italian isolates	3.01 (p<0.0001)	0.104	34.94	0.900
Italian STs	1.06 (p<0.0001)	n/a	34.91	n/a

n/a: not applicable.

Abbreviations: I_A_ Index of association values; mean dN/dS mean ratio of non-synonymous to synonymous substitutions; DI Simpson's Index of Diversity; ST Sequence Type.

**Figure 1:**
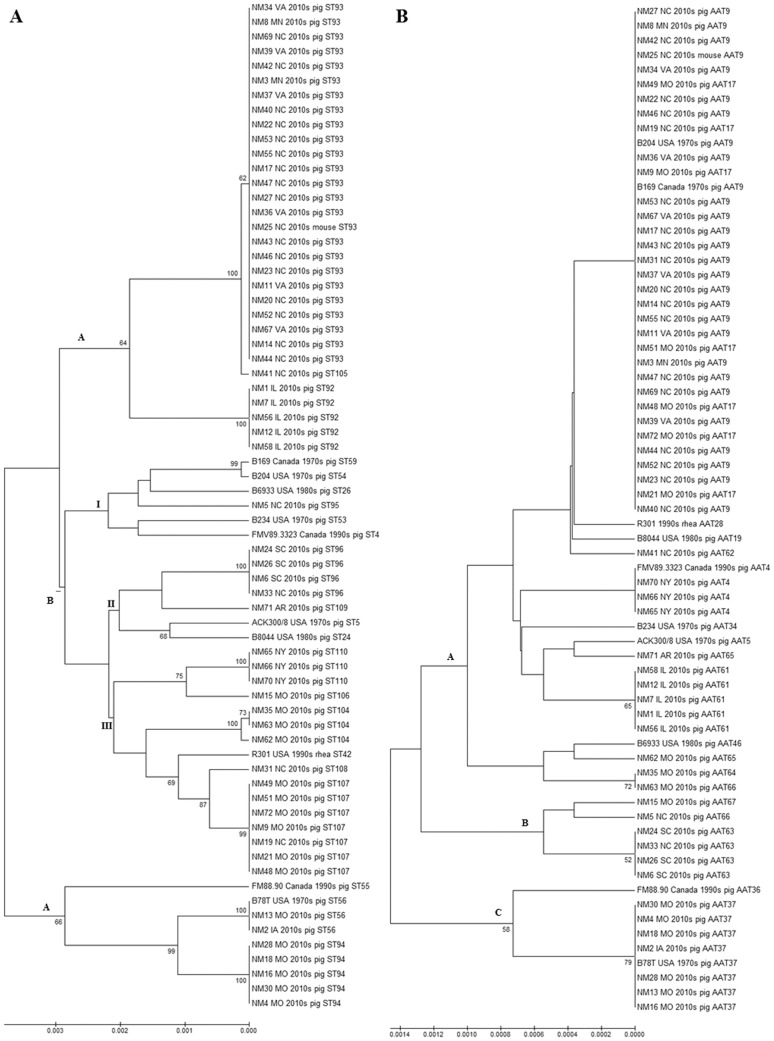
UPGMA depicting molecular relatedness of 69 North American (pre and post re-emergence) *B. hyodysenteriae* isolates. **A: Bootstrap consensus tree (1000 replicates) based on nucleotide sequences:** The Tamura 3-parameter model of Unweighted Pair Group Method with Arithmetic Mean (UPGMA) was used to compute the genetic similarity of isolates using concatenated nucleotide sequences of the seven *B. hyodysenteriae* multi-locus sequence typing loci. Bootstrap values greater than 50% are shown at the nodes, and the total length of the scale represents 30 substitutions per 10,000 base pairs of nucleotide sequence. Isolate information includes details of the ST and the state (or country), decade and host species of origin. **B: Bootstrap consensus tree (1000 replicates) based on amino acid sequences:** The dendogram was constructed and depicted as mentioned in Figure 1A; however, here amino acid sequences (AATs) were the unit of analysis. The total length of the scale represents 14 substitutions per 10,000 amino acids sequence.

The relative relationships of the 69 North American isolates based on nucleotide and amino acid differences are depicted in UPGMA dendograms (Figure 1A and Figure 1B). Based on nucleotide differences, three major lineages (denoted as A, B and C) were identified. Subgroup I of lineage B contained many historical isolates from the 1970s–1990s in addition to contemporary isolates from the 2010s, showing their relationships over time. In addition, the most divergent lineage C contained isolates from both the 1970s and 2010s characterized as ST56, showing potential conservation of nucleotides over time. Isolates and STs clustered in groups similar to the clonal complex grouping identified by the eBURST analysis. The basic topology of the dendogram with three distinct lineages (A, B and C) was similar when amino acids were used for the analysis (Figure 1B).

**Figure 2:**
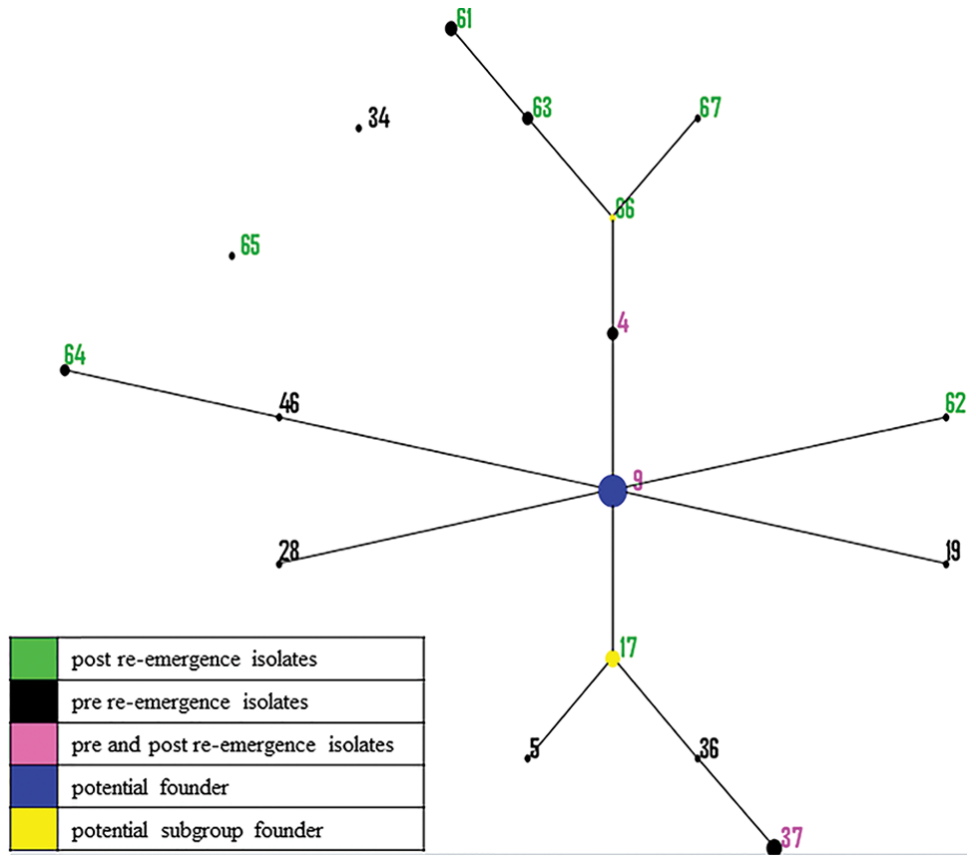
Population snapshot from 17 AATs of 69 North American isolates using the BURST algorithm. In the Based Upon Related Sequence Types (BURST) algorithm of eBURST, the labeled dots represent the amino acid type (AAT), the size of the dot denotes the number of isolates represented by that AAT, and the solid lines between two dots indicate those which are single locus variants (SLVs) of each other. The position and the distance between the dots are arbitrary. AATs that represented isolates that were classified either as pre-, post- or both (pre- and post-) re-emergence were labelled in black, green or pink, respectively. Predicted primary founder types and subgroup founder types are denoted by blue and yellow dots respectively.

The eBURST analysis used SLVs to group STs and AATs into CCs. Based on nucleotide allelic variations, 22 STs were grouped into four CCs (with two ST members each) and 14 singletons. CC11 grouped isolates originating from Canada and the U.S. in the 1970s, showing relatedness of isolates over geographical regions. CC12 grouped isolates originating from the U.S. in the 1970s and 2010s showing potential relatedness of isolates over time. Based on STs, no potential founders or subgroups were identified in the North American isolates. In contrast, based on amino acid allelic variations, the eBURST analysis grouped all but two singletons (AATs 34 and 65) into a single cluster CC1. The population snapshot analysis of the North American dataset (Figure 2), predicted a primary founder type (AAT9) that represented isolates from the 1970s (including the highly pathogenic isolate B204^R^ ATCC 31212) and 2010s (including isolates represented by the predominant strain ST93). In addition, two additional subgroups with predicted subgroup founder types (AAT17 and AAT66) were identified. In general STs were identified to be specific to a system; however ST56 was identified in two systems with a history of pig trade and movement between them. Additionally, the two systems from which ST94 were detected were identified to be located in the same geographical location, indicating a potential role of local transmission through vectors or fomites.

Similar epidemiological distributions of strains were seen in the site, system and state level when using STs and AATs as the unit of analysis. No more than one ST was found within a site, with the exception of a site that had two closely related STs (93 and 105) belonging to a common CC. In general, multiple sites owned by a common system shared a ST, with the exception of system ‘M’ (Table S1) from which four STs (56, 94, 106 and 107) were characterized, each from different sites within that system. The predominant strain ST93 was found in multiple sites in different systems. A state showed a general trend of a common ST; however, this was likely influenced by the presence of several sites belonging to a common system within a state. A common ST was found from a pig and a mouse in a system, indicating potential for transmission between the host species. In several cases, isolates from the 1970s to 2010s were represented by the same AATs, indicating likely conservation of amino acids over time.

### Global isolates

The global population of 341 isolates represented a total of 110 STs and 67 AATs based on the analysis of seven loci. Of these, 12 STs (ST92–ST96 and ST104–ST110) from the U.S. and 19 AATs (AAT49–AAT67) from Italy, the U.K. and the U.S. were newly described in this study. The most frequently isolated strains based on nucleotides included ST8, ST77 and ST93; while those based on amino acids include AAT9, AAT8, AAT59 and AAT17. The predominant strain AAT9 was identified in nine countries analyzed (Australia, Belgium, Canada, Germany, Italy, Spain, Sweden, the U.K. and the U.S.). The difference between the mean nucleotide allelic frequency of 20 and the mean amino acid allelic frequency of two indicated a high number of synonymous mutations in the population. The PubMLST database (http://pubmlst.org/bhyodysenteriae/) contains details of allele numbers, STs and AATs for all global isolates analyzed. A total of 4,110 nucleotide positions were utilized for determining the mean G+C content, the mean dN: dS ratio, the I_A_ value and the DI value (Table 1).

**Figure 3:**
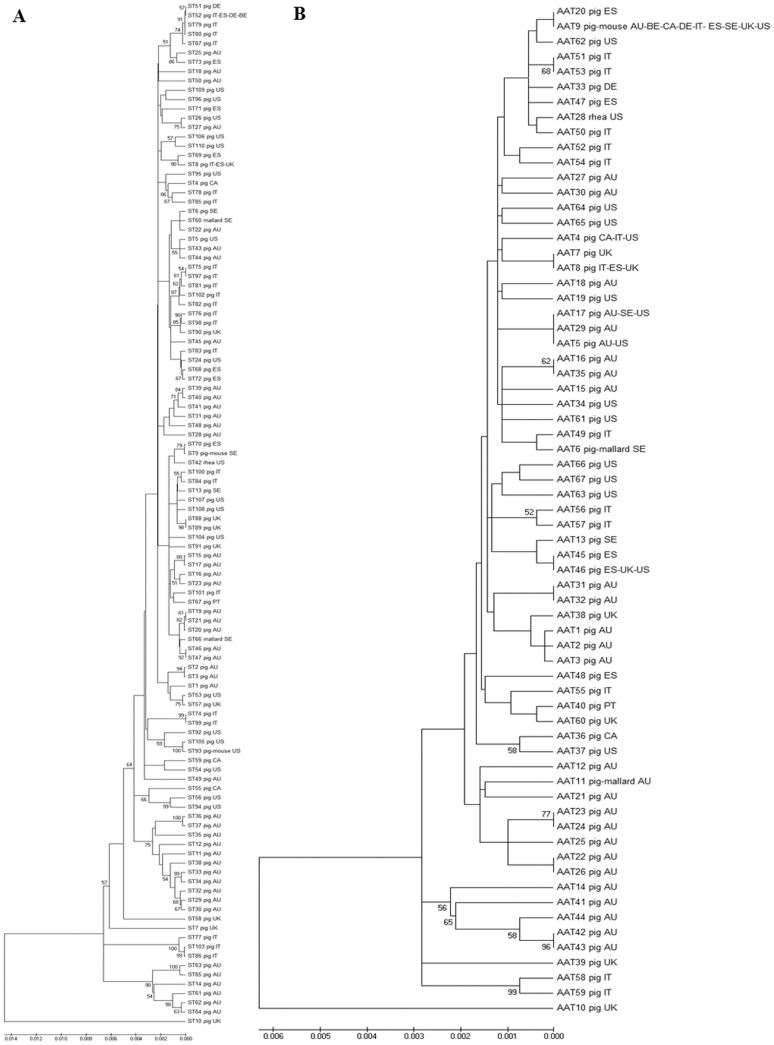
UPGMA dendograms depicting molecular relatedness of 341 global *B. hyodysenteriae* isolates. **A: Bootstrap consensus tree (1000 replicates) based on nucleotide sequences of 110 STs:** The Tamura 3-parameter model of Unweighted Pair Group Method with Arithmetic Mean (UPGMA) was used to compute the genetic similarity of isolates using concatenated nucleotide sequences of the seven *B. hyodysenteriae* multi-locus sequence typing loci. Bootstrap values greater than 50% are shown at the nodes, and the total length of the scale represents 140 substitutions per 10,000 base pairs of nucleotide sequence. ST information includes details of the host species and country (AU Australia; BE Belgium; CA Canada; DE Germany; ES Spain; IT Italy; PT Portugal; SE Sweden; UK United Kingdom; US United States of America) of origin. **B: Bootstrap consensus tree (1000 replicates) based on amino acid sequences of 67 AATs:** The dendogram was constructed and depicted as mentioned in Figure 3A, however amino acid sequences (AATs) were the unit of analysis. The total length of the scale represents 60 substitutions per 10,000 amino acid sequence.

The relationships of the 110 STs representing 341 global isolates are depicted in a UPGMA dendogram (Figure 3A). In general, clusters included different STs of isolates originating from a common country, showing a geographical relationship between the isolates and STs. In addition, STs of isolates from a common decade of isolation clustered together, showing some temporal patterns in the dendogram. Further, STs belonging to the same CC also clustered together frequently. An isolate from the U.K. representing ST10 was most distant from all other global *B. hyodysenteriae* isolates. The UPGMA analysis of 67 AATs representing 341 global isolates depicted a similar pattern of clustering of STs by geography (country of origin) and designated CC (Figure 3B).

**Figure 4:**
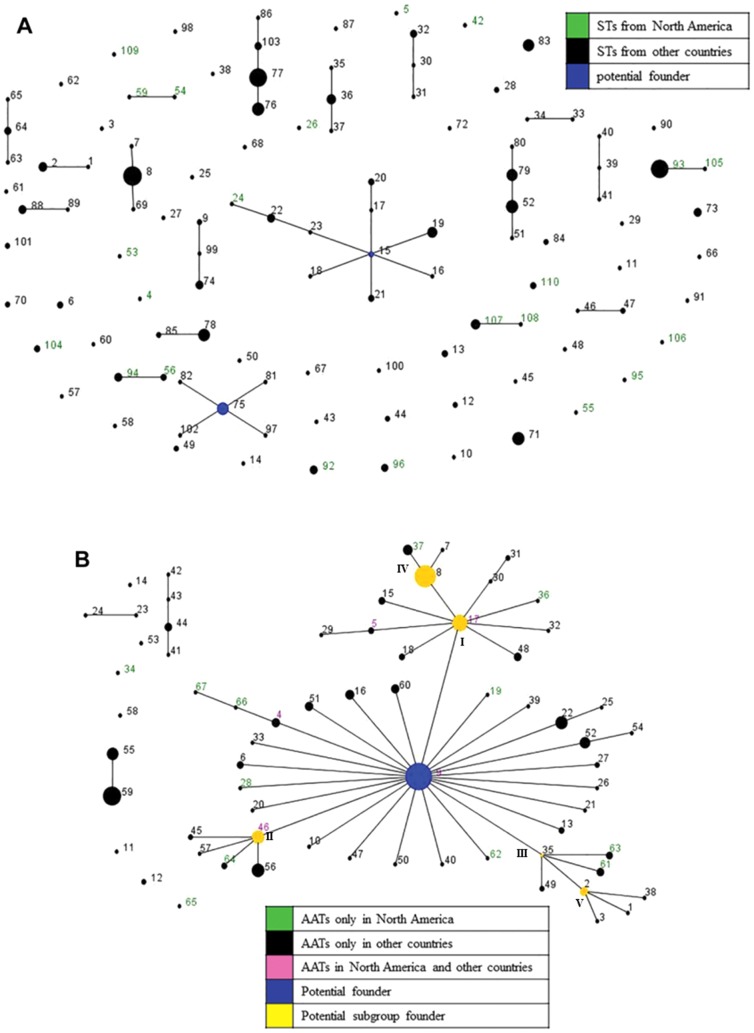
Population snapshots obtained from 341 global *B. hyodysenteriae* isolates using BURST algorithm. In the Based Upon Related Sequence Types (BURST) algorithm of eBURST, the labeled dots represent the sequence type (ST) or amino acid type (AAT) for Figure 4A and 4B, respectively; the size of the dot denotes the number of isolates represented by that ST/AAT, and the solid lines between two dots indicate those which are single locus variants (SLVs) of each other and belong to a clonal complex (CC). The position and the distance between the dots are arbitrary. **A: Based on 110 STs of the global** ***B. hyodysenteriae*** **population:** STs that represented isolates from North America or from other countries are labelled in green or black, respectively, and primary founder types are depicted by blue colored dots. **B: Based on 67 AATs of the global** ***B. hyodysenteriae*** **population:** AATs that represented isolates from North America, other countries or both are labelled in green, black or pink, respectively. Predicted primary and secondary founder types are depicted by blue and yellow colored dots respectively.

Based on nucleotide allelic profiles, the eBURST analysis identified 19 CCs and 51 singletons from the 110 STs of 341 global isolates. Although, in most cases, isolates included in a CC originated from only one country, some CCs contained isolates from more than one country within the same continent suggesting geographical relationships of strains. For instance, CCs 3, 7 and 9 included isolates from more than one European country viz. Belgium, Germany, Italy, Spain, Sweden and the U.K., and CC11 included isolates from North American countries (Canada and U.S.). CC3 included one isolate from Belgium and three isolates from Germany which were also considered to be related based on their PFGE patterns [11]. In most cases, isolates within a CC which were specific to a country originated from diverse regions or states/provinces. Out of 19 CCs, only two CCs (CC1 and CC2) were predicted to have potential founder STs (ST15 and ST75 respectively). Furthermore, several CCs contained STs of isolates originating from different decades. Interestingly, CCs 7 and 15 included STs isolated from both pigs and mice. A population snapshot of global isolates presented 19 CCs and 51 singletons, and the predicted founders ST15 [19] and ST75 of clusters CC1 and CC2 respectively (Figure 4A). No groups or subgroups with potential founders were identified from the global population as a whole, indicating a high diversity of STs.

In contrast, only four CCs and seven singletons were identified from the eBURST analysis of global isolates using SLVs of amino acid profiles. AATs of isolates originating from Australia were grouped into CCs 2 and 4, while those from Italy were grouped into CC3. The predominant CC1 included AATs of isolates from 9 countries, showing a strong epidemiological relationship of isolates based on amino acid profiles. A population snapshot analysis obtained by using AATs is presented in Figure 4B, and identifies a predominant large cluster (CC1) comprised of 52 AATs (77.62%) and 282 isolates (82.7%). Within this cluster, five subgroups (I, II, III, IV and V) were found to be linked to a primary group. The predicted primary group founder type AAT9 [18], [19] represented 62 isolates (18.18%) and 15 STs (13.64%) originating from Australia, Belgium, Canada, Germany, Italy, Spain, Sweden, U.K. and U.S. The star-like pattern depicts three subgroups viz. I, II and III of which the predicted founder types AAT17, AAT46 and AAT35, respectively, likely arose from a primary group of which AAT9 was the predicted founder. Similarly, subgroup IV (with predicted founder AAT8) and subgroup V (with predicted founder AAT2) likely arose from subgroups I and III, respectively.

**Figure 5:**
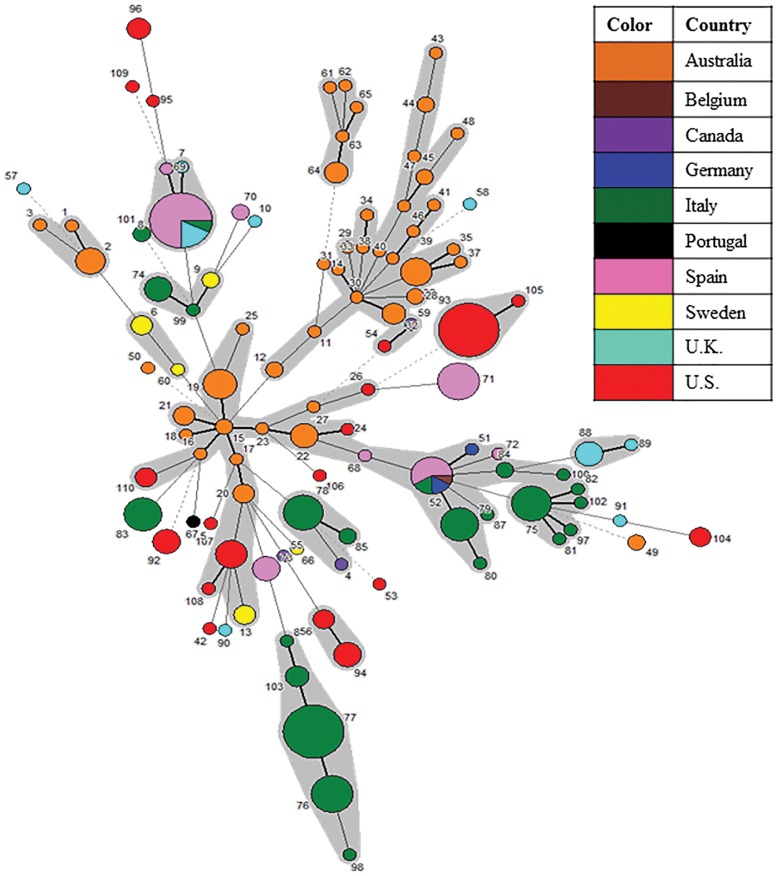
MST analysis of 110 STs representing 341 global *B. hyodysenteriae* isolates showing geographical distribution. In the Minimum Spanning Tree (MST) analysis, each circle represents a different sequence type (ST) as labeled, its size reflects the number of isolates and the color indicates the country of origin. The width of the lines reflects the genetic difference between two STs, wherein dark/heavy lines connect single locus variants (SLVs), light/thin lines connect double locus variants (DLVs) and dotted lines indicate the most likely connection between two STs differing by three or more loci. STs which were DLVs of at least one other ST were grouped together (shaded in grey).

The MST analysis based on nucleotide allelic profiles depicts the relatedness of global isolates (Figure 5). The analysis depicts the country of origin of 110 STs representing 341 global isolates grouped in CCs. Most STs were specific to the country from which the samples were isolated. ST8 represented isolates from Italy, Spain and the U.K. and ST52 represented isolates from Belgium, Germany, Italy and Spain [18], [19]. The basic clustering of STs as observed in the UPGMA dendogram and the eBURST analysis was maintained in the MST analysis. The STs tend to cluster together by geographical area (country of origin) and by time of isolation. For instance, a branch of the tree containing several ST members of CCs 1, 5, 6, 8 and 13 were isolated in the 1980s from Australia.

**Figure 6:**
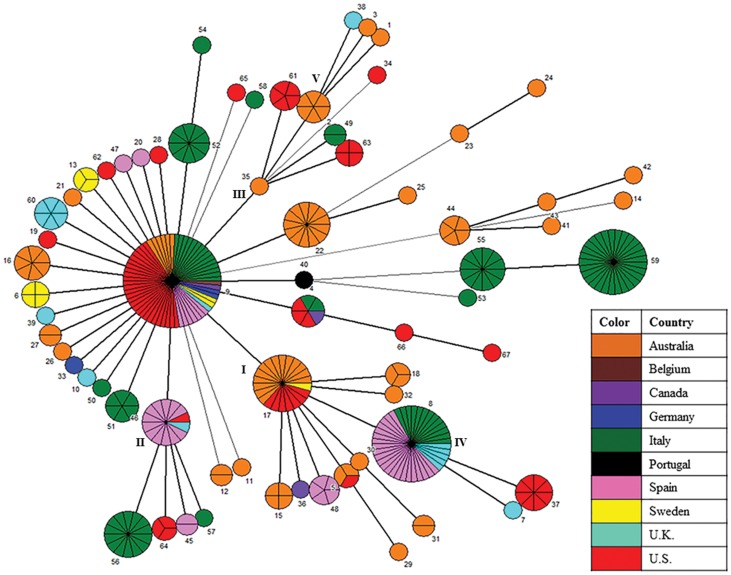
MST analysis of 67 AATs representing 341 global *B. hyodysenteriae* isolates showing geographical distribution. In the Minimum Spanning Tree (MST) analysis, each circle represents a different Amino Acid Type (AAT) as labeled, the size of the circle and the partition lines within a circle represent the relative and the absolute number of isolates represented by the AAT, and the color indicates the country of origin. The width of the lines reflects the allelic differences between two AATs, wherein dark/heavy lines connect single locus variants (SLVs) and light/thin lines connect double locus variants (DLVs).

Figure 6 illustrates the relationships and microevolution of the 341 global isolates analyzed by an MST network based on amino acid profiles. Although a majority of AATs were specific to one country, some (AATs 4, 5, 8, 9, 17 and 46) were found in more than one country. Of the six AATs found in multiple countries, four (AATs 8, 9, 17 and 46) were predicted to be primary or subgroup founders. The MST analysis showed a similar distribution of isolates and AATs as seen with the eBURST analysis wherein a predominant predicted primary founder type AAT9 representing isolates from nine countries was linked to five subgroups (I, II, III, IV and V), and predicted founders AAT8 and AAT2 of subgroups IV and V likely arose from predicted founders AAT17 and AAT35 of subgroups I and III, respectively.

In general, STs were unique to a particular country; however, two STs (ST8 and ST52) were found in more than one country within the same continent. Although in general STs clustered by a common time period of isolation, several isolates within a country were identified to share a common ST over decades, indicating the negative selective pressure acting on the loci. For instance, ST56 represented isolates originating from the U.S. in the 1970s and 2010s; ST8 represented isolates originating from the U.K. in 1990s, 2000s and 2010s; and several STs (ST74–ST79 and ST83) represented isolates originating from Italy in the 2000s and 2010s. Lastly, a predicted potential primary founder AAT9 was identified to represent pig and mouse isolates from nine countries over five decades.

## Discussion

This study analyzed the epidemiology of *B. hyodysenteriae* isolates from North America and their relationships with global isolates over the last five decades. The final dataset of 341 global isolates enabled evaluation of the population structure of *B. hyodysenteriae* and the identification of the potential founder strains. The North American isolates shared AATs with global isolates, suggesting the likelihood for a common founder type for all global *B. hyodysenteriae* isolates. The North American STs, however, were unique when compared to those of the global isolate collection, which suggests a likely independent microevolution of geographically isolated strains.

A previous MLEE study by Trott and colleagues [29] suggested that *B. hyodysenteriae* populations showed an epidemic structure wherein the populations were undergoing frequent recombination, and those strains with a selective advantage emerged as clones. These findings were contradicted by two previous MLST studies of Osorio and colleagues [18] and La and colleagues [19] which identified *B. hyodysenteriae* to have a clonal population structure. In our study, significant linkage disequilibrium (p<0.0001 for I_A_) was detected in the global *B. hyodysenteriae* population irrespective of whether the unit of analysis was isolate or ST. Significant linkage disequilibrium was also observed when populations of individual geographical areas (Australia, Italy, Spain and Portugal, and North America) were analyzed to eliminate the possibility of geographical barriers playing a role in this disequilibrium. The reasons for these contradicting results from MLEE and MLST are not clear; however, the sample set as well as the methods used for genotyping might be partially responsible for this difference. Thus, our findings of clonality in the *B. hyodysenteriae* population are consistent with those of previous MLST studies [18], [19]. In agreement with the results of previous studies [18], [19], the global and country level populations showed high DI values, indicating that *B. hyodysenteriae* populations are highly heterogeneous. In spite of the high nucleotide diversity in the global and individual country level population, the low dN: dS ratios suggested the influence of a negative or purifying selective pressure in maintaining low amino acid diversity. Locus *adh* consistently showed a higher mean G+C content when compared to the other six loci and further investigations showed that the previously designed primers [20] of gene *tdh* were incorrectly being used for the *adh* locus. To maintain consistency with previous studies, this locus is referred to as *adh* throughout this study. Furthermore, locus *adh* had the lowest number of alleles compared to the other six loci, and therefore it might be useful to use only the other six loci for future analysis.

Many epidemiologically relevant observations of the distribution of *B. hyodysenteriae* in the U.S. were made in this study. First, pigs in a herd are likely to be infected by a single strain of *B. hyodysenteriae* as indicated by the general observation of only one ST within a swine site at a single time-point. Second, ‘microevolution’ provides opportunity for the emergence of a new strain as observed in this study by the simultaneous detection of two closely related STs (93 and 105) belonging to a common CC in one swine site. ‘Microevolution’ is a process of evolution in a local population wherein new variants of a strain or species are generated in relatively shorter durations of time and it can play a crucial role in the pathogenesis of an infectious disease [30]. These findings are similar to those of previous studies [10], [19] which identified a new variant of a strain on a site which belonged to a common CC. In this instance, since ST93 was identified in multiple sites, as well as multiple isolates within the site of question, and ST105 was characterized in only one isolate from the same site, it is likely that ST105 has emerged as a new variant of the predominant ST93. Third, a common source of infection may play a role in the dissemination of *B. hyodysenteriae*, as evidenced by the identification of a common ST in different swine sites of a system. Fourth, a strain can be maintained in pig populations over long periods of time, as indicated by the detection of strain ST56 in isolates originating from the 1970s and the 2010s. Fifth, the pre re-emergent and post re-emergent *B. hyodysenteriae* isolates in the U.S. are related; as observed by the clustering of some historical isolates with those recently isolated, including ST56 and ST94. Sixth, mice can be potential reservoirs of infection on a site, as observed by the identification of a common ST in pig and mouse isolates from a swine system in this study. A previous MLEE study by Trott and colleagues [14] also detected a common strain in rodent and porcine isolates from a piggery unit, and another study by Backhans and colleagues [31] identified the close relationship of rodent and porcine *Brachyspira* spp. isolates by using species-specific PCR and sequencing. Lastly, fomites or vectors such as rodents might play a role in the transmission and spread of *B. hyodysenteriae* as observed by the occasional presence of a common ST in sites in the vicinity of each other which belong to different systems.

The epidemiological connections of strains found in North America were indicated by the temporal and geographical distribution of STs and AATs. It is possible, but not proven, that the predominant strain ST93 spread to swine herds across the country through a common source (point source or a site to site transmission) and has since been associated with those sites and systems. Additionally, it is likely that many strains were maintained in the population over several decades as implied by the identification of a common ST/AAT in isolates originating from the 1970s to 2010s, along with the detection of founder type strains.

Geographical isolation might be responsible for the unique STs identified in a country, and likely is also responsible for the detection of unique STs in North America that were not identified elsewhere globally. Trade or transport of pigs internationally is likely responsible for the instances in which STs were identified within more than one country, as observed in Europe [18]. Inherent differences in the population due to geographical isolation or insufficient sampling which failed to detect some related STs may explain the absence of a predicted primary founder ST and the high variation in the evaluated global database.

AAT9 represents a potential founder type for the North American and global population and, interestingly, also represents the predominant strain currently circulating in the U.S. It is likely that the *B. hyodysenteriae* strains currently circulating in U.S. swine herds have diversified from the historical strains in the U.S., as indicated by the observed relatedness of STs and the conservation of AATs representing pre re-emergent and post re-emergent isolates. It can be hypothesized that *B. hyodysenteriae* persisted in the U.S. without causing overt clinical signs over the last few decades, and the current re-emergence is associated with a resurgence of clinical signs (either due to increased virulence or decreased antimicrobial susceptibility of the pathogen), and consequently increased diagnostic laboratory submissions and increased awareness of the disease. The high likelihood of AAT9 being a founder type is supported by its consistent presence in nine countries over five decades (1970s to 2010s) and in multiple host species (pig and mouse).

There are several strengths to MLST as a genotyping and molecular epidemiological tool for *B. hyodysenteriae*. Nucleotide differences (STs) are used for identifying strains due to its high discriminatory power, and therefore have proven to be very useful for molecular epidemiological and surveillance studies. However, due to the relatively high diversity of *B. hyodysenteriae* based on nucleotides, STs have limited utility to study potential microevolutionary relationships. Amino acids of these same loci show a much higher conservation, likely due to the negative selection of any mutations that might disrupt the function of these housekeeping genes, and therefore better visualizes the potential microevolutionary relationships of the evaluated isolates. The amino acid types (AATs) are therefore useful for studying the potential microevolution and divergence of *B. hyodysenteriae*. Another advantage of MLST is that it can be used to generate an expandable, accessible and fully portable electronic database with relevant information of evaluated isolates, proving very useful for global epidemiological studies. STs could potentially also be used to identify or track the spread of strains with a character of interest such as lowered susceptibility towards an antimicrobial. The newly analyzed isolates from the U.S. in this study were all obtained from diagnostic submissions of clinical cases and therefore it is possible that some strains currently circulating in the U.S. are not represented in this study. However, the isolates were selected from major swine systems and swine rearing states, and therefore likely represent the general strain diversity in the U.S. Finally, MLST may or may not have the high resolution required to track the spread of an outbreak in real time as it progresses; however, it is useful for long-term epidemiological studies and potential microevolutionary studies of *B. hyodysenteriae*.

This is the first study to characterize the strains of *B. hyodysenteriae* and study its epidemiology in the U.S. after its re-emergence. These epidemiological analyses emphasize the importance of biosecurity measures to prevent the spread of *B. hyodysenteriae* to and between swine sites. It also highlights the utility of MLST as a tool for routine genotyping and in studying the epidemiology and microevolution of *B. hyodysenteriae* on the local, national and global scale.

## Supporting Information

Table S1 Epidemiological and genotypic information for 69 North American B. hyodysenteriae isolates evaluated in this study.
(DOCX)
