# Supplementary material for: Understanding the Molecular Epidemiology and Global Relationships of Brachyspira hyodysenteriae from Swine Herds in the United States: A Multi-Locus Sequence Typing Approach
Source: PLoS One. 2014 Sep 5;9(9):e107176. doi: 10.1371/journal.pone.0107176 (PMC4156428; doi:10.1371/journal.pone.0107176)
Supplement: Table S1 — Epidemiological and genotypic information for 69 North American B. hyodysenteriae isolates evaluated in this study. (DOCX) [file pone.0107176.s001.docx]

**Table S1: Epidemiological and genotypic information for 69 North American *B. hyodysenteriae* isolates evaluated in this study.**

| **Isolate** | **Country** | **State** | **System** | **Site** | **Decade** | **Host** | ***adh* (nt)** | ***alp* (nt)** | ***est* (nt)** | ***gdh* (nt)** | ***glpK* (nt)** | ***pgm* (nt)** | ***thi* (nt)** | **ST** | **CC_(ST)_** | **AAT** | **CC_(AAT)_** |
| --- | --- | --- | --- | --- | --- | --- | --- | --- | --- | --- | --- | --- | --- | --- | --- | --- | --- |
| ACK300/8 | USA | unkw. | unkw. | unkw. | 1970s | pig | 2 | 10 | 3 | 10 | 8 | 1 | 3 | 5 | * | 5 | 1 |
| B8044 | USA | unkw. | unkw. | unkw. | 1980s | pig | 2 | 12 | 3 | 4 | 8 | 2 | 3 | 24 | 1 | 19 | 1 |
| B6933 | USA | unkw. | unkw. | unkw. | 1980s | pig | 2 | 16 | 5 | 4 | 6 | 3 | 3 | 26 | * | 46 | 1 |
| R301 | USA | unkw. | unkw. | unkw. | 1990s | rhea | 2 | 11 | 8 | 5 | 9 | 2 | 10 | 42 | * | 28 | 1 |
| B234 | USA | unkw. | unkw. | unkw. | 1970s | pig | 2 | 6 | 7 | 2 | 4 | 2 | 7 | 53 | * | 34 | * |
| B204 | USA | unkw. | unkw. | unkw. | 1970s | pig | 1 | 16 | 3 | 4 | 2 | 3 | 6 | 54 | 11 | 9 | 1 |
| B78T | USA | unkw. | unkw. | unkw. | 1970s | pig | 2 | 2 | 1 | 10 | 5 | 1 | 11 | 56 | 12 | 37 | 1 |
| NM2 | USA | IA | I | I1 | 2010s | pig | 2 | 2 | 1 | 10 | 5 | 1 | 11 | 56 | 12 | 37 | 1 |
| NM13 | USA | MO | M | M3 | 2010s | pig | 2 | 2 | 1 | 10 | 5 | 1 | 11 | 56 | 12 | 37 | 1 |
| NM7 | USA | IL | E | E1 | 2010s | pig | 2 | 6 | 17 | 5 | 26 | 3 | 3 | 92 | * | 61 | 1 |
| NM1 | USA | IL | E | E2 | 2010s | pig | 2 | 6 | 17 | 5 | 26 | 3 | 3 | 92 | * | 61 | 1 |
| NM56 | USA | IL | E | E2 | 2010s | pig | 2 | 6 | 17 | 5 | 26 | 3 | 3 | 92 | * | 61 | 1 |
| NM58 | USA | IL | E | E2 | 2010s | pig | 2 | 6 | 17 | 5 | 26 | 3 | 3 | 92 | * | 61 | 1 |
| NM12 | USA | IL | E | E3 | 2010s | pig | 2 | 6 | 17 | 5 | 26 | 3 | 3 | 92 | * | 61 | 1 |
| NM3 | USA | MN | C | C1 | 2010s | pig | 2 | 16 | 14 | 24 | 26 | 3 | 13 | 93 | 15 | 9 | 1 |
| NM27 | USA | NC | D | D1 | 2010s | pig | 2 | 16 | 14 | 24 | 26 | 3 | 13 | 93 | 15 | 9 | 1 |
| NM17 | USA | NC | D | D2 | 2010s | pig | 2 | 16 | 14 | 24 | 26 | 3 | 13 | 93 | 15 | 9 | 1 |
| NM69 | USA | NC | F | F1 | 2010s | pig | 2 | 16 | 14 | 24 | 26 | 3 | 13 | 93 | 15 | 9 | 1 |
| NM20 | USA | NC | F | F2 | 2010s | pig | 2 | 16 | 14 | 24 | 26 | 3 | 13 | 93 | 15 | 9 | 1 |
| NM44 | USA | NC | F | F3 | 2010s | pig | 2 | 16 | 14 | 24 | 26 | 3 | 13 | 93 | 15 | 9 | 1 |
| NM46 | USA | NC | F | F3 | 2010s | pig | 2 | 16 | 14 | 24 | 26 | 3 | 13 | 93 | 15 | 9 | 1 |
| NM47 | USA | NC | F | F3 | 2010s | pig | 2 | 16 | 14 | 24 | 26 | 3 | 13 | 93 | 15 | 9 | 1 |
| NM23 | USA | NC | F | F4 | 2010s | pig | 2 | 16 | 14 | 24 | 26 | 3 | 13 | 93 | 15 | 9 | 1 |
| NM34 | USA | VA | H | H1 | 2010s | pig | 2 | 16 | 14 | 24 | 26 | 3 | 13 | 93 | 15 | 9 | 1 |
| NM11 | USA | VA | H | H2 | 2010s | pig | 2 | 16 | 14 | 24 | 26 | 3 | 13 | 93 | 15 | 9 | 1 |
| NM37 | USA | VA | H | H2 | 2010s | pig | 2 | 16 | 14 | 24 | 26 | 3 | 13 | 93 | 15 | 9 | 1 |
| NM39 | USA | VA | H | H2 | 2010s | pig | 2 | 16 | 14 | 24 | 26 | 3 | 13 | 93 | 15 | 9 | 1 |
| NM36 | USA | VA | H | H3 | 2010s | pig | 2 | 16 | 14 | 24 | 26 | 3 | 13 | 93 | 15 | 9 | 1 |
| NM8 | USA | MN | J | J1 | 2010s | pig | 2 | 16 | 14 | 24 | 26 | 3 | 13 | 93 | 15 | 9 | 1 |
| NM52 | USA | NC | L | L1 | 2010s | pig | 2 | 16 | 14 | 24 | 26 | 3 | 13 | 93 | 15 | 9 | 1 |
| NM53 | USA | NC | L | L1 | 2010s | pig | 2 | 16 | 14 | 24 | 26 | 3 | 13 | 93 | 15 | 9 | 1 |
| NM55 | USA | NC | L | L1 | 2010s | pig | 2 | 16 | 14 | 24 | 26 | 3 | 13 | 93 | 15 | 9 | 1 |
| NM25 | USA | NC | L | L2 | 2010s | mouse | 2 | 16 | 14 | 24 | 26 | 3 | 13 | 93 | 15 | 9 | 1 |
| NM40 | USA | NC | L | L3 | 2010s | pig | 2 | 16 | 14 | 24 | 26 | 3 | 13 | 93 | 15 | 9 | 1 |
| NM42 | USA | NC | L | L3 | 2010s | pig | 2 | 16 | 14 | 24 | 26 | 3 | 13 | 93 | 15 | 9 | 1 |
| NM43 | USA | NC | L | L3 | 2010s | pig | 2 | 16 | 14 | 24 | 26 | 3 | 13 | 93 | 15 | 9 | 1 |
| NM22 | USA | NC | L | L4 | 2010s | pig | 2 | 16 | 14 | 24 | 26 | 3 | 13 | 93 | 15 | 9 | 1 |
| NM14 | USA | NC | L | L5 | 2010s | pig | 2 | 16 | 14 | 24 | 26 | 3 | 13 | 93 | 15 | 9 | 1 |
| NM67 | USA | VA | H | H4 | 2010s | pig | 2 | 16 | 14 | 24 | 26 | 3 | 13 | 93 | 15 | 9 | 1 |
| NM4 | USA | MO | B | B1 | 2010s | pig | 2 | 2 | 1 | 10 | 26 | 1 | 11 | 94 | 12 | 37 | 1 |
| NM28 | USA | MO | M | M1 | 2010s | pig | 2 | 2 | 1 | 10 | 26 | 1 | 11 | 94 | 12 | 37 | 1 |
| NM30 | USA | MO | M | M2 | 2010s | pig | 2 | 2 | 1 | 10 | 26 | 1 | 11 | 94 | 12 | 37 | 1 |
| NM16 | USA | MO | M | M4 | 2010s | pig | 2 | 2 | 1 | 10 | 26 | 1 | 11 | 94 | 12 | 37 | 1 |
| NM18 | USA | MO | M | M9 | 2010s | pig | 2 | 2 | 1 | 10 | 26 | 1 | 11 | 94 | 12 | 37 | 1 |
| NM5 | USA | NC | P | P1 | 2010s | pig | 2 | 2 | 19 | 4 | 27 | 3 | 3 | 95 | * | 66 | 1 |
| NM33 | USA | NC | K | K1 | 2010s | pig | 2 | 25 | 19 | 4 | 7 | 3 | 13 | 96 | * | 63 | 1 |
| NM6 | USA | SC | K | K2 | 2010s | pig | 2 | 25 | 19 | 4 | 7 | 3 | 13 | 96 | * | 63 | 1 |
| NM26 | USA | SC | K | K3 | 2010s | pig | 2 | 25 | 19 | 4 | 7 | 3 | 13 | 96 | * | 63 | 1 |
| NM24 | USA | SC | K | K4 | 2010s | pig | 2 | 25 | 19 | 4 | 7 | 3 | 13 | 96 | * | 63 | 1 |
| NM62 | USA | MO | A | A1 | 2010s | pig | 2 | 26 | 5 | 4 | 28 | 2 | 24 | 104 | * | 64 | 1 |
| NM63 | USA | MO | A | A1 | 2010s | pig | 2 | 26 | 5 | 4 | 28 | 2 | 24 | 104 | * | 64 | 1 |
| NM35 | USA | MO | A | A1 | 2010s | pig | 2 | 26 | 5 | 4 | 28 | 2 | 24 | 104 | * | 64 | 1 |
| NM41 | USA | NC | L | L3 | 2010s | pig | 2 | 16 | 20 | 24 | 26 | 3 | 13 | 105 | 15 | 62 | 1 |
| NM15 | USA | MO | M | M5 | 2010s | pig | 2 | 2 | 19 | 4 | 6 | 2 | 25 | 106 | * | 67 | 1 |
| NM48 | USA | MO | M | M6 | 2010s | pig | 2 | 11 | 8 | 10 | 6 | 2 | 11 | 107 | 14 | 17 | 1 |
| NM49 | USA | MO | M | M6 | 2010s | pig | 2 | 11 | 8 | 10 | 6 | 2 | 11 | 107 | 14 | 17 | 1 |
| NM51 | USA | MO | M | M6 | 2010s | pig | 2 | 11 | 8 | 10 | 6 | 2 | 11 | 107 | 14 | 17 | 1 |
| NM72 | USA | MO | M | M7 | 2010s | pig | 2 | 11 | 8 | 10 | 6 | 2 | 11 | 107 | 14 | 17 | 1 |
| NM9 | USA | MO | M | M8 | 2010s | pig | 2 | 11 | 8 | 10 | 6 | 2 | 11 | 107 | 14 | 17 | 1 |
| NM21 | USA | MO | M | M10 | 2010s | pig | 2 | 11 | 8 | 10 | 6 | 2 | 11 | 107 | 14 | 17 | 1 |
| NM19 | USA | NC | N | N1 | 2010s | pig | 2 | 11 | 8 | 10 | 6 | 2 | 11 | 107 | 14 | 17 | 1 |
| NM31 | USA | NC | O | O1 | 2010s | pig | 2 | 11 | 8 | 24 | 6 | 2 | 11 | 108 | 14 | 9 | 1 |
| NM71 | USA | AR | Q | Q1 | 2010s | pig | 2 | 27 | 3 | 25 | 29 | 3 | 13 | 109 | * | 65 | * |
| NM65 | USA | NY | G | G1 | 2010s | pig | 2 | 2 | 3 | 5 | 6 | 2 | 11 | 110 | * | 4 | 1 |
| NM66 | USA | NY | G | G1 | 2010s | pig | 2 | 2 | 3 | 5 | 6 | 2 | 11 | 110 | * | 4 | 1 |
| NM70 | USA | NY | G | G1 | 2010s | pig | 2 | 2 | 3 | 5 | 6 | 2 | 11 | 110 | * | 4 | 1 |
| FMV89.3323 | Canada | unkw. | unkw. | unkw. | 1990s | pig | 2 | 3 | 3 | 1 | 4 | 3 | 16 | 4 | * | 4 | 1 |
| FM88.90 | Canada | unkw. | unkw. | unkw. | 1990s | pig | 2 | 11 | 1 | 10 | 6 | 1 | 9 | 55 | * | 36 | 1 |
| B169 | Canada | unkw. | unkw. | unkw. | 1970s | pig | 1 | 16 | 3 | 4 | 2 | 3 | 7 | 59 | 11 | 9 | 1 |

“*” indicates singletons for either nucleotides or amino acids.

Abbreviations: unkw. unknown; nt nucleotide allele; ST Sequence Type; CC_(ST)_ Clonal Complex of Sequence Type; AAT Amino Acid Type; CC_(AAT)_ Clonal Complex of Amino Acid Type.
